# Scale development and validation of social media mindfulness: evidence from Chinese Douyin users

**DOI:** 10.3389/fpsyg.2026.1660076

**Published:** 2026-06-12

**Authors:** Mengyu Li, Hao Chen

**Affiliations:** 1 School of Journalism and Communication, Henan University of Technology, Zhengzhou, China; 2 School of Film Television and Communication, Xiamen University of Technology, Xiamen, China

**Keywords:** Douyin users, empirical validation, mental health, scale development, social media mindfulness

## Abstract

Mindfulness has been widely conceptualized and measured across various fields. With increasing attention to the healthy usage of social media, the present study aims to develop a measurement scale for social media mindfulness, intended to guide users towards purposeful and intentional usage, thereby alleviating the adverse effects on mental health caused by obsessive or addictive use of social media. Two studies, comprising the development of the measurement scale and validation through a nomological network linking social media mindfulness and users’ psychological wellbeing, were conducted. Consequently, four factors encompassing 19 measuring items were synthesized which are nonjudgement (6 items), attention-acceptance (6 items), description (4 items) and nonreaction (3 items), respectively. Furthermore, the influence of four subconstructs of social media mindfulness on Douyin users’ psychological wellbeing has been further validated to ensure the criterion-related validity of the developed measurement scale. Consequently, all constructs have been examined to be positively related to users’ psychological wellbeing. Overall, the developed scale offers foundation for future research concerning mindful social media usage. More importantly, mindfulness-based intervention designed to mitigate the negative effects of social media usage can be constructed based on the scale’s dimensions.

## 1 Introduction

With the rapid development of 5G technology and mobile applications, social media platforms have undergone a significant growth, with an increasing number of people engaging in and using social media every day (Brailovskaia and Margraf, 2024; Weaver and Swank, 2024). Therefore, a substantial body of research has been conducted to investigate the impacts of social media and mobile applications on various aspects of people’s lives (Bartosik-Purgat et al., 2017; Karim et al., 2020). There is no denying that the use of social media can generate positive and enjoyable outcomes, but it is also associated with mental health issues such as depression, anxiety, and loneliness (Sadagheyani and Tatari, 2021; Ulvi et al., 2022), especially among younger generations due to addictive use of diverse platforms such as Twitter, Instagram, TikTok, and others across the globe (Abi-Jaoude et al., 2020; Berryman et al., 2018; Naslund et al., 2020). Mindfulness, which has been defined as increased attention to and non-judgemental awareness to the present moment, is positively correlated with self-awareness of ones’ own behavior and self-efficacy (Brailovskaia and Margraf, 2022; Gilbert and Waltz, 2010; Wilmer et al., 2017). Therefore, to promote the positive use of social media, mindfulness-based interventions (MBIs) have been proposed to help users engage with social media in a mindful and constructive way, thereby ameliorating the negative outcomes associated with feelings of exclusion on social media (Jones et al., 2022; Poon and Jiang, 2020).

At present, alternative method have been proposed to measure mindfulness. While mindfulness is generally measured using self-assessment instruments designed to accommodate different conceptualizations and operationalizations, these tools have been psychometrically validated (Sauer et al., 2013). Specifically, within the family of psychometric scales used to assess mindfulness, the Cognitive and Affective Mindfulness Scale Revised (CAMS-R) (Feldman et al., 2007), Developmental Mindfulness Survey (DMS) (Solloway and Fisher, 2007), Effects of Mediation Scale (EOM) (Reavley and Pallant, 2009), Five Factors Mindfulness Questionnaire (FFMQ) (Baer et al., 2006), and Mindfulness Attention Awareness Scale (MAAS) (Brown and Ryan, 2003; Höfling et al., 2011), among others, were developed based on different definitions and application contexts. The case most closely related to the present study is the measurement scale proposed by Shabahang et al. (2024) which concern the mindful use of social media. However, their study included only seven items designed to assess mindful social media use within a single subdimension. Thus, the above-mentioned scales serve as the theoretical scaffold to guide the contextual adaptation of mindfulness to the specific setting of social media use. In this vein, the proposed scale was understood as a domain-specific operationalization of mindful social media engagement, in which only those facets that remain conceptually distinct and behaviorally meaningful in social media contexts are expected to emerge as stable dimensions.

Naturally, the present study aims to develop a more comprehensive, multi-dimensional measurement scale to evaluate social media users’ mindfulness in relation to their usage, following rigorous procedures recommended by Churchill (1979). Different from the general daily activities, social media use occurs in a technologically mediated environment designed to maximize engagement through features such as algorithmic personalization, intermittent rewards (e.g., likes, comments, notifications), infinite scroll, rapid content switching, and social evaluative cues. These features to a great extent can intensify attentional capture, habitual checking, emotional reactivity, and social comparison (Fournier et al., 2026; Yue et al., 2022).

Usually, trait mindfulness reflects a general tendency toward mindfulness that applies to a wide range of contexts, from work to personal relationships, and does not necessarily address the specific features or demands for capturing the distinct challenges posed by social media environments (Jones et al., 2022; Mesmer-Magnus et al., 2017).

The connection between users’ mindfulness and their mental health in the context of social media usage has been well investigated by prior studies (Brailovskaia and Margraf, 2022; Harvey and Aikman, 2025; Sun, 2023), with users’ mindfulness acting as a mediating or moderating variable in the correlation between different patterns of social media use or psychological factors and mental health outcomes (Flett et al., 2019; Jones et al., 2022; Moqbel et al., 2024). However, a systematic approach to measuring mindfulness in relation to social media use remains underdeveloped, as prior studies have typically examined social media users’ mindfulness using pre-existing general mindfulness scales. Therefore, by applying the modified procedure for measurement development suggested by Churchill (1979), the present study aims to develop a measurement scale for social media mindfulness, based on evidence from Douyin users in Mainland China.

Moreover, the relationship between users’ social media mindfulness and their psychological wellbeing will be empirically examined to validate the proposed measurement scale of social media mindfulness based on the requirement of criterion-related validity. This potential relationship has been built based on the previous studies. For example, Chen et al. (2024) have explored the effect of mindfulness across the levels of interpersonal, group, organization, media and cross-culture communication. Specifically, it has been concluded that integrating mindfulness principles into digital and media design can create more engaging, restorative experiences that support mental health and wellbeing. Besides, Liu et al. (2025b) have empirically determined that mindfulness meditation led by short videos significantly reduced individual’s communication anxiety and mood disorders which implies that to some extent, it can be an accessible tool for enhancing mental wellbeing. Thus, by establishing a reliable and valid measurement scale, this study may pave the way for mindfulness-based interventions that promote more mindful use of social media, which, in turn, could contribute to preventing mental health issues among social media users.

Accordingly, the development of social media mindfulness measurement scale in this study could offer a foundational research framework for the future investigations into the application of mindfulness in social media contexts. This is especially significant in the current era, as it may help to prevent mental health disorders among social media users and ultimately support the sustainable development and improved service quality of social media platforms. That is, this validated mindfulness scale, tailored specifically to social media, can inform platform designers and policymakers by guiding the development of more user-centric, ethically designed digital spaces. Subsequently, it could serve as the foundation for the sustainable development of both social media and society at large. To date, psychometric approach based on participants’ self-reported perceptions has been the primary method for evaluating mindfulness, owing to its convenience, ease of application, and well-established empirical support (Baer et al., 2004; Bohlmeijer et al., 2010; Sauer et al., 2013). Thus, this study will develop a social media domain of mindfulness during individual’s social media engagement by using a psychometric approach based on a self-reported questionnaire.

## 2 Literature review

### 2.1 Social media usage and mindfulness

There is no denying that people now live in a technology-driven world, where internet browsing and social media usage have become salient aspects people’ life (Shabahang et al., 2024). Social media provides access to needed information, offers platforms for self-expression, and facilitates connections with others to satisfy the human need for social presence (Han et al., 2015; Kietzmann et al., 2011). However, with the rapid development of these platforms, an increasing number of users have reported becoming obsessed with them, and this problematic behavior has negatively impacted their personal lives, work, and academic pursuits (Chatterjee, 2021; Sun, 2022; Wallace, 2014;). Diverse studies from multiple perspectives have sought to address these concerns, including peer support groups (Tan et al., 2021), emotion regulation strategies (Chirico et al., 2024), short-term abstinence interventions (Zhou et al., 2021), among others.

Among different strategies to cope with the compulsive or addictive use of social media, mindfulness which is considered as “an openhearted, moment-to-moment non-judgmental awareness” has been postulated as a significant adaptive strategy to mitigate the negative impacts associated with social media use (Kabat-Zinn, 2023). Numerous studies have confirmed the significance of mindfulness in enhancing users’ awareness and intentional engagement with social media, thereby reducing the likelihood of mental health issues, such as anxiety and depression caused by the excessive usage or the fear of missing out (Baker et al., 2016; Kostanski and Hassed, 2008; Sofia et al., 2023). As Rheingold (2012) suggests, using social media mindfully resembles meditation, in the sense that when users become aware that their attention has started to drift, the mindfulness they possess helps gently redirect their focus to the present moment. That is, individuals with higher levels of mindfulness tend to engage with social media deliberately and purposefully and intentionally, which to a large extent, helps prevent them from becoming absorbed in the digital world (Bhatia and Sethi, 2024; Kaminska, 2023).

### 2.2 Social media mindfulness and mental health

As a multifaceted construct, mindfulness has been conceptualized and measured across various disciplines and from different perspectives, and the definition of mindfulness has consistently been complex and heterogeneity (Alvear et al., 2022; Sauer et al., 2013). In the context of social media, instruments such as FFMQ, MAAS, DMS or Kentucky Inventory of Mindfulness Skills (KIMS) have been widely employed to assess users’ mindfulness, often with certain adaptations to suit the research context (i.e., Charoensukmongkol, 2016; Jones et al., 2022; Kim et al., 2023). More recently, to ensure greater applicability within the social media domain, Shabahang et al. (2024) introduced the concept of mindful use of social media and developed the Mindful Use of Social Media Scale (MUSMS), drawing upon data from 676 active users in Iran and the USA. This scale comprises seven items measured on a six-point Likert scale and provides a robust framework for operationalizing social media mindfulness in the current study. More importantly, the existing body of knowledge has suggested that mindfulness-based intervention could lead to an increased active use of social media, during which users remain aware of their environment, sensations, thoughts, and feelings. This, in turn, may play a protective role in mitigating the adverse influences of social media use, such as addiction (Du et al., 2023), and may also have a positive impact on users’ psychological wellbeing (Hanley et al., 2015) or life satisfaction (Christopher and Gilbert, 2010; Li et al., 2022).

## 3 Research methodology

**Figure 1:**
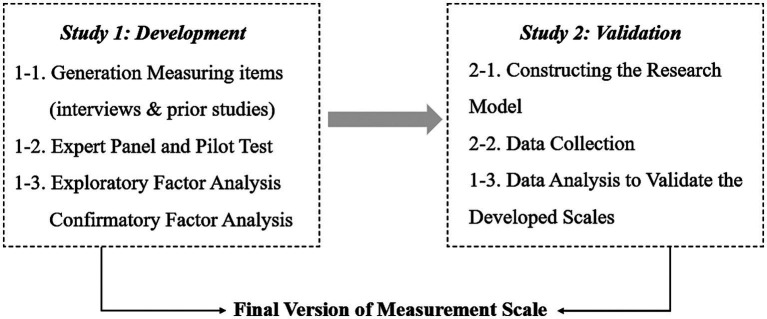
Research design for the development and validation of the measurement scale.

To develop a measurement scale for social media mindfulness and empirically validate it, two major studies were conducted to jointly achieve the research objectives. Particularly, Study 1 focuses on the development of the social media mindfulness measurement scale, based on the revised procedures recommended by Churchill (1979). Study 2 concentrates on validating the developed scale by empirically investigating the relationship between social media mindfulness and social media users’ psychological wellbeing (Hanley et al., 2015; Hinkin, 1995; Lomas et al., 2017; Walsh et al., 2007). More importantly, the designed questionnaire and the semi-structured interview questions have been approved by the Academic Committee of School of Journalism and Communication, Henan University of Technology (No. HAUTIRB-2025-0609). Furthermore, informed consent has been obtained from all participants, and they have been notified that the collected data will remain anonymous and will be used exclusively for academic purposes, with no involvement in any commercial activities. The processes of scale development and validation are synthesized in Figure 1, based on the procedures outlined by Churchill (1979) and Zhou (2019).

Since the data collection was to be conducted in China, the questionnaire, initially designed in English, was translated into Chinese to accommodate Chinese respondents. The back-translation method was applied in this study to ensure the accuracy and appropriateness of the questions (Kivela et al., 1999). The final English version of the questionnaire was translated into Chinese by a professional bilingual translator fluent in both in English and Chinese. Subsequently, the Chinese version was translated back into English by another professional bilingual proficient in both languages. Finally, the researchers and both translators reviewed the Chinese version together to verify for the development and validation of the measurement scale.

### 3.1 Study 1: development of measurement scale of social media mindfulness

#### 3.1.1 Generating measurement items

Based on the suggestion provided Churchill (1979), several methods could be used to generate an original pool of measurement items, including literature search, experience survey, insight-stimulating examples, critical incidents and focus groups. Since several measurement scales of mindfulness have been developed in diverse contexts, the literature search was used as the initial method to gather potential items for measuring social media mindfulness.

Afterwards, semi-structured interviews were conducted both online and offline from 10 October 2024 to 5 November 2024, to explore users’ mindfulness in relation to their social media use. The general questions posed during the interviews focused on questions such as “How much do you know about the mindfulness?,” “When you use social media, do you use it intentionally and purposively?,” “T*o what extent do you think that you can control yourself when you use social media?,*” and “What kind of thoughts do you have when you use social media?.” Moreover, the researcher flexibly guided the discussion, probing further based on the informants’ responses to elicit broader insights and perspectives (Solloway and Fisher, 2007).

Finally, to capture the broad concept of users’ social media mindfulness, informants of different genders (10 females and 8 males), ages (ranging from 18 to 55 years old), and occupations (students, institutional staff, businesspeople, etc.) were recruited. All participants had experience using Douyin, one of the most popular social media platforms in China (Fitzgerald et al., 2022; Zhao and Ye, 2025). The interview was ended until there is no new content generated based on the theoretical saturation (Moura et al., 2021). As results, a total of 18 informants was interviewed, with each interview lasting approximately 40 min, and an initial dataset of 18,000 Chinese characters was recorded. Thematic analysis was applied to extract key themes from the recorded interview data, following the procedures of grounded theory (Chapman et al., 2015). Specifically, the author has conducted the coding process and the other two researchers were recruited to evaluate the coding results. Discussion and corresponding revisions have been performed until the final agreement has been made among them. Finally, based on the findings from semi-structured interviews, and in conjunction with the literature review, a total of 32 initial measuring items were generated.

#### 3.1.2 Expert panel and pilot test

A draft questionnaire was designed based on the 32 measurement items obtained from the above-discussed procedures (Wang et al., 2018). The statements relating to social media mindfulness were measured by a seven-point Likert Scale, with 1 representing “never or very rarely true” to 7 representing “always or almost always true” (Dundas et al., 2013). Subsequently, a panel of five experts familiar with the mindfulness and social media were recruited to assess the applicability of the measurement items and the design of the questionnaire, to improve its overall quality. Furthermore, a small sample for conducting a pilot test was selected through a purposive sampling method before the formal data collection which consists of 35 participants with experience using Douyin. Overall, followed the procedures by previous studies, the early scale development stages were intended primarily for item generation and refinement rather than formal qualitative or psychometric evaluation. Accordingly, the expert review and pilot test in this stage were the developmental procedures used to improve item clarity and contextual relevance (Churchill, 1979; Yadav and Rahman, 2017). In summary, no duplicated items were found among the initial 32 measurement items, although some nuanced revisions were made to facilitate comprehension in relation to the phrasing of the measurement items. Consequently, the final version of the questionnaire was finalized for formal data collection and analysis.

#### 3.1.3 Final survey

The developed measurement scale was validated using data collected from an online panel study (Callegaro et al., 2014; Porter et al., 2019; Wang et al., 2018). A purposive sampling method was adopted to gather relevant data from Douyin users by Credamo,^1^ a platform that provides scientific questionnaire data services to scholars across more than 1,800 universities worldwide (Tang et al., 2023; Wu et al., 2022). Data collection was conducted between 15 December 2024 and 5 January 2025, spanning approximately three weeks. Initially, 823 responses were obtained; however, 76 responses were excluded due to issues such as straight-lining (i.e., giving identical responses across all items), insufficient response time, and missing data. Consequently, 747 valid responses, representing a validity rate of 90.77%, were retained for formal data analysis. According to the sample size recommendations proposed by Cochran (1977), this final sample was deemed adequate and was subsequently divided into two groups for conducting exploratory factor analysis (EFA) and confirmatory factor analysis (CFA), respectively.

### 3.2 Study 2: validation of measurement scale of social media mindfulness

Following the initial development of the social media mindfulness measurement scale, the nomological network framework was employed to further validate the scale (Cronbach and Meehl, 1955). As outlined in section 2.2, the reliability and validity of the scale were further assessed to evaluate its predictive power in identifying the association between the various dimensions of social media mindfulness and users’ mental health. In this context, users’ psychological wellbeing was selected as the criterion variable (Evli et al., 2023; Sun, 2023). Moreover, the measurement items of social media users’ psychological wellbeing were applied from the work of Diener et al. (2009), and a total of five items were used to estimate users’ psychological wellbeing in terms of self-esteem, sense of purpose, and interpersonal relationships.

Data collection was also carried out by Credamo, with the process taking place between 5 February and 20 February 2025. Ultimately, a total of 298 valid responses, representing a validity rate of 92.26%, were derived from an initial sample of 323 participants and were used to examine the nomological network of the developed measurement scale. The data were analyzed using structural equation modelling, conducting through AMOS 29.0. The statistics of measurement and structural models are presented below to demonstrate the nomological validity of the measurement scale (Cronbach and Meehl, 1955).

## 4 Results

### 4.1 Results of study 1

#### 4.1.1 Socio-demographic characteristics, normality

Among the valid 747 samples, 398 were females, accounting for 53.28% of the total, and 349 were male, representing 46.72%. This gender distribution closely aligns with the user profile of Douyin in 2024 (iiMedia Research, 2025). Moreover, the average age of respondents was 27.34 years, which falls within the predominant user age group of 18–35 years, as indicated by Nanjing Marketing Group (2025). Moreover, over half of the respondents (389 individuals; 52.07%) held or were pursuing a bachelors’ degree, followed by 234 individuals (31.33%) with a high school diploma or a three-year college qualification. The majority of respondents reported a monthly income between 8,000 and 12,999 CNY (307 individuals, 41.1%). Based on the IP address data, the respondents primarily came from Beijing (157 individuals, 21.02%), Shanghai (114 individuals, 15.26%), Guangdong province (107 individuals, 14.32%), Zhejiang province (87 individuals, 11.65%) and other provinces such as Henan, Jiangsu, and Sichuan.

Typically, factor analysis is influenced by the distribution patterns in the data, especially in cases of deviation from multivariate normality. Therefore, normality tests for the variables were conducting using SPSS 29.0. Kurtosis and skewness values between −2 to +2 were treated as indicative of approximate normality (Mardia, 1970). Based on the results, the kurtosis and skewness values of the collected data fell within the recommended thresholds, revealing that the data were normally distributed. Consequently, subsequently factor analysis using AMOS 29.0 version was deemed appropriate. Moreover, the dataset was divided into two groups: a calibration sample and validation sample (Galvao et al., 2005), using the random function in SPSS 29.0. The calibration sample (*n* = 374) was used to identify latent factors and assess the effectiveness of the measurement scale through EFA, while the validation sample (*n* = 373) was utilized to determine the overall model fit, as well as the reliability and validity of the scale, via CFA.

#### 4.1.2 Results of exploratory factor analysis

The calibration sample of 374 responses were used to conduct the EFA to uncover the underlying structure of the dataset by identifying relationships among multiple variables (Schmitt, 2011). The Kaiser-Meyer-Olkin (KMO) measure of sampling adequacy was found to be 0.918, and Bartlett’s test of sphericity was statistically significant (*p* < 0.001), suggesting that the calibration sample data were suitable for EFA (Bonacich, 1972; Field, 2013).

Measuring items of social media mindfulness were refined based on the following criterion recommended, as recommended by Field (2013) and Kaiser (1974). Items with factor loadings lower than 0.4 were removed, items loading onto two factors simultaneously with both loadings higher than 0.4 were also eliminated, and items whose removal did not significantly reduce the reliability of the scale based on the values of “Cronbach’s alpha if item was deleted” and the internal reliability of the factors were excluded. The EFA was repeated until all refined items met the specified criteria (Wang et al., 2018). Consequently, 19 measuring items, grouped into four factors, were retained from the original pool of 32 items. Moreover, the four factors had eigenvalues values higher than 1.0, and the factor loadings of the items were found to be above 0.450 (Gould, 1967). In addition, the four extracted four factors explained 52.76% of the variances, exceeding the minimum threshold of 50% suggested by Hair et al. (2009). Finally, all factors demonstrated Cronbach’s alpha values greater than 0.7, indicating strong internal consistency (Hair et al., 2009; Taber, 2018).

Table 1 presents the results of the extracted factors and their corresponding measurement items from the calibration sample (*n* = 374). The first factor, termed as nonjudgement, comprises six measurement items and accounts for 34.765% of variance (*α* = 0.798), aligning with the conceptualization of Baer et al. (2006). The second factor, labelled awareness-acceptance, includes six measurement items, explains 8.488% of the variance, and has a reliability coefficient of α = 0.802. Subsequently, the third factor, named description, consists of four measurement items, accounting for 5.437% of variance (α = 0.812). Finally, the fourth factor, referred to as nonreaction, contains three measuring items relevant to the social media context, accounting for 4.072% of variance with the reliability of α = 0.792. The labelling of each factor was independently conducted by two researchers, and the final agreement was reached collaboratively. The naming of each factor was primarily based on the nature of the measurement items included within the construct.

**Table 1:** Results of exploratory factor analysis (*n* = 374 of calibration sample).

Factors/items	Factor loadings	Eigen-values	% of variance	CA
Factor 1: nonjudgment (NJ)		8.612	34.765	0.798
NJ1: I think some of my emotions are bad or inappropriate and I should not feel them when I use Douyin^.a^	0.762			
NJ2: I use Douyin without being aware that I am using it.	0.489			
NJ3: I use Douyin automatically without evaluating about how worthwhile or worthless my experiences are.	0.654			
NJ4: I approve myself when I have attritional ideas about the content posted on Douyin.	0.563			
NJ5: I do not control my feelings when I have the feeling about the content on Douyin.	0.768			
NJ6: I tend to judge whether my perceptions are right or wrong.^a^	0.564			
Factor 2: attention-Acceptance (AA)		1.778	8.488	0.802
AA1: When I am using Douyin, I only focus on Douyin instead of thinking of others.	0.623			
AA2: I find myself using Douyin without paying attention^.a^	0.660			
AA3: When I use Douyin, my attention is on Douyin only.	0.622			
AA4: When I using Douyin, my mind is tied to Douyin and it is not easy to distract me.	0.647			
AA5: I use Douyin purposely with attention on Douyin.	0.542			
AA6: I tend to do other things altogether with using Douyin rather than just watching Douyin at a time.^a^	0.555			
Factor 3: description (D)		1.456	5.437	0.812
D1: I can easily find the words to describe my feelings and post my reviews on Douyin.	0.618			
D2: I am good at finding the words to express my feeling on Douyin.	0.604			
D3: When I have a sensation about the content posted on Douyin, I can describe it with the appropriate words	0.787			
D4: It is hard for me to find the words to express my feeling to the content I watch on Douyin.^a^	0.620			
Factor 4: Nonreaction (NR)		1.028	4.072	0.792
NR1: I perceive my feelings and emotions without having to react to them.	0.488			
NR2: I can watch my feeling without getting lost them when I use Douyin.	0.670			
NR3: When I am using Douyin I tend to daydream inspired from posts and contacts from Douyin.^a^	0.702			

CA, Cronbach’s alpha. ^a^Present the reverse-coded items (1 = 7, 2 = 6, 3 = 5).

#### 4.1.3 Results of confirmatory factor analysis

The CFA was conducted to test the reliability and validity of the developed measurement model using a validation sample of 373 participants. The construct reliability was evaluated using Cronbach’s alpha, with a threshold value set as 0.7. Convergent and discriminant validity were assessed through CFA (Churchill, 1979), using the average variance extracted (AVE) and the square root of the AVE compared to the inter-construct correlation matrix (Fornell and Larcker, 1981; Kline, 2015). The model’s goodness-of-fit indices were reported as χ^2^/df = 2.113, CFI = 0.923, TLI = 0.932 and RMSEA = 0.042. Based on established thresholds, all indices met the recommendation criterion, namely CFI > 0.9, TLI > 0.9, χ^2^/df < 5, RMSEA<0.05 (Hair et al., 2009; Kline, 2015), suggesting an acceptable model fit as shown in Tables 2, 3.

**Table 2:** Results of confirmatory factor analysis (*n* = 373 of validation sample).

Factors/items	Factor loadings	CA	CR	AVE
Factor 1: nonjudgment (NJ)		0.832	0.843	0.475
NJ1	0.662			
NJ2	0.589			
NJ3	0.754			
NJ4	0.673			
NJ5	0.802			
NJ6	0.632			
Factor 2: attention-acceptance (AA)		0.875	0.896	0.592
AA1	0.711			
AA2	0.812			
AA3	0.732			
AA4	0.778			
AA5	0.765			
AA6	0.814			
Factor 3: description (D)		0.844	0.865	0.616
D1	0.744			
D2	0.780			
D3	0.802			
D4	0.812			
Factor 4: Nonreaction (NR)		0.812	0.808	0.584
NR1	0.779			
NR2	0.738			
NR3	0.774			

CR, Cronbach’s alpha; CR, Composite Reliability; AVE, Average Variance Extracted.

**Table 3:** The discriminant validity results (*n* = 373 of validation sample).

Factors	AVE	NJ	AA	D	NR
NJ	0.475	0.689			
AA	0.592	0.692**	0.769		
D	0.616	0.497**	0.606**	0.785	
NR	0.584	0.602**	0.534**	0.512**	0.764

Figures in diagonal line are the square rooted AVE. **represents significance at 0.01 level.

Specifically, Table 2 presents the results of the conducted on a validation sample of 373 participants. As demonstrated in the table, all factor loadings within each construct were found to be greater than 0.5, ranging from 0.589 to 0.814. Furthermore, the Cronbach’s alpha values all exceeded 0.7, and the composite reliability (CR) were above 0.8 (NJ: α = 0.832, CR = 0.843; AA: α = 0.875, CR = 0.896; D: α = 0.844, CR = 0.865; NR: α = 0.812, CR = 0.808). Moreover, the AVE values, indicating construct validity, were above 0.5 for three constructs (AA: AVE = 0.592; D: AVE = 0.616; NR: AVE = 0.584). Only the NJ factor, with an AVE value of 0.475, was marginally acceptable for convergent validity, as its value is above the threshold of 0.36 (Fornell and Larcker, 1981).

In detail, the first factor comprises items such as “I think some of my emotions are bad or inappropriate and I shouldn’t feel them when I use Douyin” (NJ1) and “I use Douyin automatically without evaluating about how worthwhile or worthless my experiences are” (NJ3) which are similar to the concept of nonjudging of inner experience proposed by the FFMQ. “Nonjudging” refers to adopting a non-evaluative and accepting attitude towards one’s thoughts and feelings. In the context of social media usage, this implies that users do not adopt an evaluative stance towards their experience of using social media, especially Douyin in this case (Ortet et al., 2020). The second factor, which includes items such as “When I am using Douyin, I only focus on Douyin instead of thinking of others” (AA1) and “When I using Douyin, my mind is tied to Douyin and it is not easy to distract me” (AA4), was labelled “awareness-acceptance.” Based on the content of FFMQ, one facet of acting with awareness involves paying attention to one’s activities at the moment, as opposed to operating on autopilot and allowing one’s attention to drift elsewhere. Moreover, it has been suggested that mindfulness is the combination of present-moment awareness and acceptance of internal experiences, rather than simply the ability to observe one’s mental processes. The integration of awareness and acceptance has been previously discussed (Cardaciotto, 2005). The third factor, which includes four items such as “I can easily find the words to describe my feelings and post my reviews on Douyin” (D1) and “When I have a sensation about the content posted on Douyin, I can describe it with the appropriate words” (D2), was labelled “description,” as this dimension refers to users’ ability to identify and articulate their internal experiences using appropriate language. Finally, the “nonreaction” factor, with measuring items such as “I perceive my feelings and emotions without having to react to them” (NR1) and “*I can watch my feeling without getting lost them when I use Douyin* (NR2),” was identified and labelled accordingly, as the items were loaded strongly on this construct and closely aligned with the tendency to allow thoughts and feelings to come and go without getting carried away by them (Baer et al., 2008). The current finding is broadly consistent with the FFMQ; however, the observing factor which refers to noticing or attending to internal and external experiences such as cognitions, emotions or sense perception is absent. Statistically, although observing-related items were initially included in the item pool, they were later deleted due to low factor loadings and cross-loadings on multiple factors.

In addition to the reliability and convergent validity, discriminant validity was evaluated using a matrix of correlation coefficients and the square root of the AVE. According to Kline (2015), discriminant validity is considered acceptable when the square root of factor’s AVE exceeds its correlation coefficients with other factors. Table 3 presents the results of discriminant validity results. As shown in the table, the square root values of AVE for three factors are higher than their respective correlation coefficients, with the exception of the correlation between nonjudgment and attention-acceptance, which was recorded at 0.692 which is slightly higher than the square root of the AVE value of 0.689. Thus, the discriminant validity was generally acceptable, although the distinction between nonjudgment and attention-acceptance should be interpreted with caution due to their high correlation and the marginal AVE of nonjudgment. Moreover, all correlation coefficients fall within the range of 0.3–0.7, indicating appropriate relationships between factors within the same construct as well as adequate distinctions among different factors. Therefore, the discriminant validity of the developed measurement scale for social media mindfulness is supported.

### 4.2 Results of study 2

The nomological network was applied to examine the nomological validity of the proposed measurement scale for social media mindfulness within a broader framework. Cronbach and Meehl (1955) emphasized that constructs should be situated within a nomological system and exhibit logically consistent relationships with related concepts within the framework. Establishing nomological validity is a crucial stage in scale development, serving to evaluate the construct validity of the scale. As discussed in section 2.2 and 3.2, a total 298 responses were utilized to examine the nomological validity by establishing the connections between social media mindfulness and users’ psychological wellbeing.

Among the valid 298 samples, 159 females were female (53.36%) and 139 were males (46.44%). The average age of respondents was 26.47 years, which closely aligns with the collected data in Study 1. Moreover, approximately two-thirds (298 respondents) were either holders of a bachelor’s degree or currently pursuing one. The majority of respondents (177 individuals, 59.4%) reported a monthly income between 8,000 and 12,999 CNY. Based on the IP address data, the respondents primarily came from Beijing, Shanghai, Guangdong province (107 individuals, 14.32%), Zhejiang province (87 individuals, 11.65%) and other provinces such as Shandong, Jiangsu, and Sichuan.

As results, it was found that no single component explains more than 50% of the covariance with 37.25% of the variance from the first component based on the Harman one-factor test, suggesting there is no issue concerning the common method bias (Podsakoff et al., 2003). Moreover, the goodness-of fit indices indicated an acceptable level with χ^2^/df = 2.253, CFI = 0.945, TLI = 0.950 and RMSEA = 0.037. Moreover, the analysis confirmed that all these four factors which are nonjudgement (*β* = 0.464, *p* < 0.001), attention-acceptance (*β* = 0.456, *p* < 0.001), description (*β* = 0.396, *p* < 0.001) and nonreaction (*β* = 0.337, *p* < 0.001), are significantly and positively related to the social media users’ psychological wellbeing. Thus, the developed measurement model was further validated.

## 5 Conclusion and discussion

### 5.1 Discussion

The present study developed and validated a Social Media Mindfulness Scale (SMMs) based on empirical data collected from Douyin users in China. First, a sample of 747 responses was used to calibrate and validate the development of the measurement scale. Subsequently, a separate set of 298 responses was employed to examine the nomological validity of the SMMS and to further verify the developed measurement scale. As results, the final scale comprised 19 items across four major dimensions which are Nonjudgment (NJ), Attention-Acceptance (AA), Description (D), and Nonreaction (NR), respectively. The findings contribute to the growing body of research on mindfulness in digital contexts by offering a psychometrically validated tool for assessing how users engage with social media mindfully. In particular, this study contributes to the existing body of knowledge from the following perspectives.

In terms of the implications for theoretical development, previous mindfulness scales (e.g., FFMQ, MAAS, etc.) were developed for general psychological contexts (Baer et al., 2006; Brown and Ryan, 2003). Recent research relevant to social media contexts have typically adapted these existing scales to examine issues within the realm of social media usage (i.e., Moqbel et al., 2024; Wang et al., 2025). However, social media introduces unique challenges, such as compulsive scrolling, fear of missing out, obsessive engagement, and emotional regulation, which necessitate a context-specific mindfulness measurement. Thus, the development and validation of the SMMs help bridge this gap by reconceptualizing traditional mindfulness dimensions in relation to social media behaviors, ensuring that the scale captures platform-specific patterns of attention and emotional regulation (Kabat-Zinn, 2003; Liu et al., 2023). The items within each subconstruct have been validated specifically in the context of social media use. Accordingly, the SMMS may serve as a foundational framework for future research on mindfulness in social media contexts.

Moreover, the procedures for developing and validating the measurement scale in this study may serve as a framework for future research on scale construction, as this study strictly adhered to the steps proposed by Churchill (1979). More importantly, the nomological network analysis recommended by Cronbach and Meehl (1955) was conducted to establish nomological validity which provides a research paradigm for future research concerning the measurement development. Finally, this study has examined the relationship between users’ mindful engagement with social media and their psychological wellbeing, offering a nuanced understanding that goes beyond general mindfulness traits. This may contribute to future research on mindfulness-based interventions targeting unhealthy social media usage. Additionally, the study by Mattes (2019) indicated that non-judging, as a subconstruct of mindfulness, had the highest impact on avoiding undesirable outcomes, according to a meta-analysis. This aligns closely with the findings of the current study, implicitly suggesting that greater emphasis should be placed on nonjudgmental interventions in the context of social media.

At last, one of the most important findings highlighted by this study is that, compared to the FFMQ, which is the most commonly used measurement scale for mindfulness, this study validates four subdimensions for assessing users’ mindfulness in the context of social media, excluding the “observing” facet (Mattes, 2019). This may be attributed to the specific context of the present study, social media usage which is strongly associated with outcomes related to users’ mental health issues. While the study by Rudkin et al. (2018) revealed that the observing facet of FFMQ exhibited unexpected relationships with psychological symptoms, MUSMS proposed by Shabahang et al. (2024), which comprises seven items, also omitted any reference to the observing facet. This implicitly suggests that the FFMQ may lack generalizability within the context of social media mindfulness. Thus, the developed measurement scale by the present study may, to a great extent, lay a theoretical foundation for the measurement of social media mindfulness for the future research related to individual’s mindful usage of social media.

As for the practical implications provided by the present study, the SMMS offers valuable applications for multiple stakeholders. First, mental health professionals specializing in social media usage can apply the scale to identify the individual exhibiting problematic social media use patterns, particularly those scoring low in non-reactivity or non-judgemental tendencies because the significance of these two dimensions are positively associated with Douyin users’ psychological wellbeing. Furthermore, their description or awareness can also be measured to evaluate individuals’ levels of social media mindfulness, which are correlated to their mental health. Timely adaptive strategies could then be implemented. This is very in line with the finding suggested by Liu et al. (2025a) that mental health professionals could incorporate mindfulness techniques derived from short-term monasticism into their therapeutic practices, helping clients develop better self-awareness and coping strategies. Moreover importantly, unlike some studies on the mindful use of social media (Shabahang et al., 2024) that treat the concept as a single construct, the scale’s specific dimensions allow for targeted interventions, such as attention-training exercises for users with difficulties in focus or emotion-regulation strategies for those displaying high reactivity. Social media platforms themselves could integrate the scale’s principles into their design, potentially incorporating features such as mindfulness prompts, usage dashboards, or algorithm adjustments that promote more intentional engagement. Finally, educational institutions may incorporate the SMMS into digital literacy programs to foster healthier online habits among students and other digital active users, which is a significant social concern in today’s digital world. The strong psychometric properties and demonstrated the relationship with psychological wellbeing underscore the scale’s potential as both a research tool and clinical instrument.

### 5.2 Limitations and suggestions for future research

Overall, this study develops a measurement scale for mindfulness in the context of social media, which is significant to social media development and provides adaptive psychological strategies for users to engage with social media while reducing negative outcomes, such as mental health disorders. However, this study has some limitations. First, the pool of measurement was derived from existing literature and the analytical results of semi-structured interviews. However, other qualitative research methods, such as focus group or online reviews analytics, could be employed to facilitate the extraction of initial measuring items pool. Second, building on the existing mindfulness measurement scales such as MAAS, FFMQ, and EOM, this study developed its scale based on participants’ self-reported cognitive assessments of their mindfulness levels. Though this approach offers certain advantages, other approaches such as monitoring brainwaves using electroencephalograms or tracking eye movements could be applied simultaneously to capture social media users’ mindfulness both psychometrically and physically. Finally, differences in cultural and educational backgrounds as well as gender or age, which might influence users’ mindfulness related to social media, was not considered, as the primary aim of this study was to develop a measurement scale specific to social media mindfulness (Khoury et al., 2013; Mattes, 2019). The dataset used in the study was drawn from socially and digitally developed regions, such as Beijing and Shanghai. Thus, the generalizabity of the findings should be approached with caution. That is, the present study should be understood as providing initial validation evidence within a predominantly young adult, urban-leaning sample, rather than definitive evidence of applicability across the full Douyin user population. Future research could explore differences in social media mindfulness across socio-demographic characteristics, such as gender, age, regions such as rural or urban areas and so forth.

## Data Availability

The raw data supporting the conclusions of this article will be made available by the authors, without undue reservation.

## Footnotes

1
www.credamo.com
